# A novel inorganic–organic hybrid borate, poly{[Na_2_(C_4_H_2_O_4_)(H_3_BO_3_)(H_2_O)_4_]·H_3_BO_3_}

**DOI:** 10.1107/S1600536810041358

**Published:** 2010-10-23

**Authors:** Zhi-Dong Shao, Yu-Qi Zhang, Shu-Li Wu, Yun-Xiao Liang

**Affiliations:** a State Key Laboratory Base of Novel Functional Materials and Preparation Science, Faculty of Materials Science and Chemical Engineering, Ningbo University, Ningbo, Zhejiang, 315211, People’s Republic of China

## Abstract

The structure of the title compound, *catena*-poly[[[di-μ-aqua-μ-fumarato-μ-(boric acid)-disodium]-di-μ-aqua] boric acid monosolvate], contains two crystallographically independent Na^+^ cations, each being six-coordinated by one fumarate O atom, one boric acid O atom and four water O atoms in a distorted octa­hedral geometry. Adjacent [NaO_2_(OH_2_)_4_] units share edges and are linked into chains propagating parallel to [100]. The free boric acid mol­ecules are connected to the chains through strong inter­molecular O—H⋯O hydrogen bonds. Additional O—H⋯O hydrogen bonds between the water mol­ecules, the free and coordinated boric acid mol­ecules and the fumarate anion lead to the formation of a three-dimensional supra­molecular structure. With the exception of the two water mol­ecules, all other atoms lie on mirror planes.

## Related literature

For the synthesis of organic ammonium borates, see: Li *et al.* (2006 ▶); Wang *et al.* (2004 ▶); Liu *et al.* (2008 ▶). For the synthesis of metal borates with neutral amines, see: Sung *et al.* (2000 ▶); Zhang *et al.* (2004 ▶); Liu *et al.* (2006 ▶); Wang *et al.* (2005 ▶). For borates involving organic acids, see: Tombul *et al.* (2007 ▶); Wu *et al.* (2009 ▶). For typical Na—O bond lengths, see: Yi *et al.* (2005 ▶); Huang *et al.* (2005 ▶); for B—O bond lengths, see: Li *et al.* (1999 ▶); Andrews *et al.* (1983 ▶); Roy *et al.* (2002 ▶).
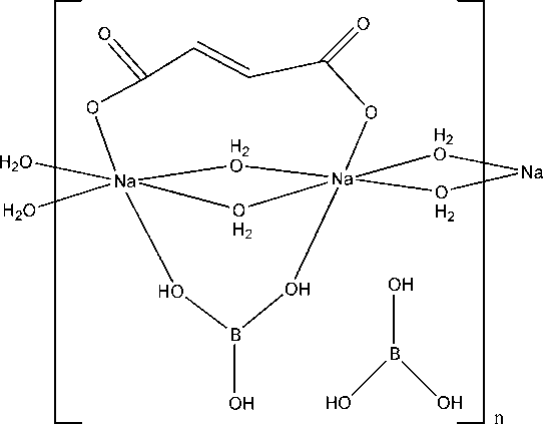


## Experimental

### 

#### Crystal data

[Na_2_(C_4_H_2_O_4_)(H_3_BO_3_)(H_2_O)_4_]·H_3_BO_3_
*M* *_r_* = 355.77
Orthorhombic, 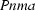

*a* = 14.116 (3) Å
*b* = 6.9347 (14) Å
*c* = 14.997 (3) Å
*V* = 1468.1 (5) Å^3^
*Z* = 4
Mo *K*α radiation
μ = 0.21 mm^−1^
*T* = 295 K
0.39 × 0.26 × 0.25 mm

#### Data collection

Rigaku R-AXIS RAPID diffractometer
Absorption correction: multi-scan (*ABSCOR*; Higashi, 1995 ▶) *T*_min_ = 0.924, *T*_max_ = 0.950
13772 measured reflections
1806 independent reflections
1460 reflections with *I* > 2σ(*I*)
*R*_int_ = 0.023

#### Refinement

*R*[*F* ^2^ > 2σ(*F* ^2^)] = 0.036
*wR*(*F* ^2^) = 0.103
*S* = 1.10
1806 reflections
127 parameters
H-atom parameters constrained
Δρ_max_ = 0.48 e Å^−3^
Δρ_min_ = −0.23 e Å^−3^

### 

Data collection: *RAPID-AUTO* (Rigaku, 1998 ▶); cell refinement: *RAPID-AUTO*; data reduction: *CrystalStructure* (Rigaku/MSC, 2002 ▶); program(s) used to solve structure: *SHELXS97* (Sheldrick, 2008 ▶); program(s) used to refine structure: *SHELXL97* (Sheldrick, 2008 ▶); molecular graphics: *ORTEPII* (Johnson, 1976 ▶) and *DIAMOND* (Brandenburg & Putz, 2008 ▶); software used to prepare material for publication: *SHELXL97*.

## Supplementary Material

Crystal structure: contains datablocks I, global. DOI: 10.1107/S1600536810041358/wm2409sup1.cif

Structure factors: contains datablocks I. DOI: 10.1107/S1600536810041358/wm2409Isup2.hkl

Additional supplementary materials:  crystallographic information; 3D view; checkCIF report

## Figures and Tables

**Table:** 

Na1—O5	2.3756 (17)
Na1—O1	2.3771 (17)
Na1—O12	2.4140 (12)
Na1—O11	2.4529 (12)
Na2—O6	2.3606 (16)
Na2—O12^i^	2.4353 (12)
Na2—O11	2.4541 (12)
Na2—O4	2.5727 (17)
B1—O7	1.357 (3)
B1—O5	1.364 (3)
B1—O6	1.366 (3)
B2—O8	1.348 (3)
B2—O10	1.372 (2)
B2—O9	1.373 (2)

**Table:** 

O7—B1—O5	123.60 (18)
O7—B1—O6	119.43 (18)
O5—B1—O6	116.97 (18)
O8—B2—O10	120.84 (18)
O8—B2—O9	122.36 (17)
O10—B2—O9	116.80 (17)

Symmetry code: (i) 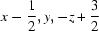.

**Table 2:** Hydrogen-bond geometry (Å, °)

*D*—H⋯*A*	*D*—H	H⋯*A*	*D*⋯*A*	*D*—H⋯*A*
O6—H6*A*⋯O8	0.90	1.79	2.690 (2)	177
O10—H10*A*⋯O7	0.84	1.86	2.6973 (19)	178
O5—H5*A*⋯O4^ii^	0.88	1.80	2.6713 (19)	177
O7—H7*A*⋯O3^ii^	0.87	1.74	2.6104 (18)	178
O12—H12*B*⋯O3^iii^	0.95	2.04	2.9355 (15)	157.8
O12—H12*A*⋯O9^iv^	0.80	2.02	2.8063 (14)	171.5
O11—H11*B*⋯O2^iii^	0.82	1.98	2.8042 (14)	175.5
O11—H11*A*⋯O10^iv^	0.90	2.24	3.0494 (15)	150.5
O8—H8*A*⋯O1^i^	0.86	1.79	2.6559 (19)	180
O9—H9*A*⋯O2^i^	0.84	1.82	2.6504 (19)	168

Symmetry codes: (i) 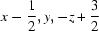; (ii) 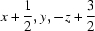; (iii) 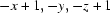; (iv) 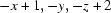.

**Fig. 1.**
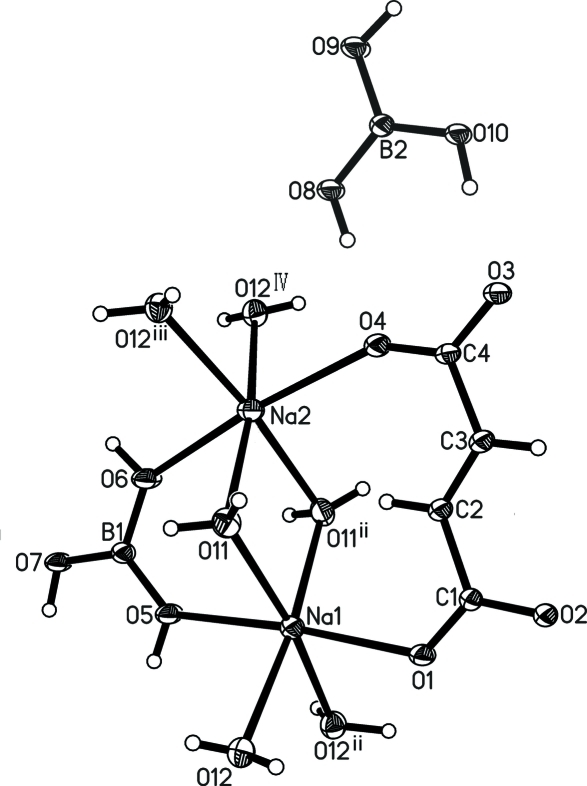
The molecular structure of the title compound with the atomic labelling. Displacement ellipsoids are drawn at the 30% probability level and all H atoms are shown as small spheres of arbitrary radius. [Symmetry codes: (ii) x, -y+1/2, z; (iii) x-1/2, y, -z+3/2; (iv) x-1/2, -y+1/2, -z+3/2.]

**Fig. 2.**
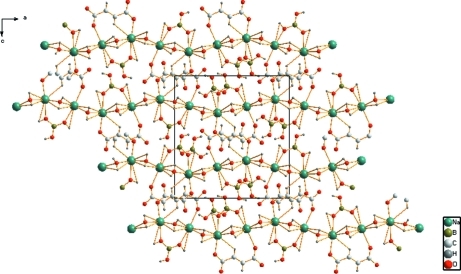
A representation of the one-dimensional infinite chain propagating parallel to [100] in the structure of (I).

**Fig. 3.**
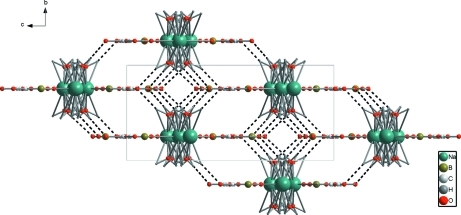
The three-dimensional supramolecular network in the structure of (I).

## supplementary crystallographic information

### Comment

Borates have attracted great attention owing to their rich structural chemistry and important technical applications. Borate materials with various main group, rare earths and transition metals have been widely explored. In contrast, less work has been carried out on inorganic-organic hybrid borates. Up to date, only a few organic amines have been successfully introduced in their cationic forms into borate systems, such as [NH_3_CH_2_CH_2_NH_3_][B_6_O_9_(OH)_2_] (Li *et al.*, 2006), [H_3_N(C_6_H_10_)NH_3_][B_4_O_5_(OH)_4_] and [H_3_N(C_6_H_10_)NH_3_][B_5_O_8_(OH)] (Wang *et al.*, 2004) and [C_6_H_13_N_2_][B_5_O_6_(OH)_4_] (Liu *et al.*, 2008), or metals coordinated by neutral amines, such as [Cu(en)_2_][B_7_O_13_H_3_]_n_ (en is ethylenediamine; Sung *et al.*, 2000), [Mn(C_10_H_18_N_6_)][B_5_O_6_(OH)_4_]_2_ (Zhang *et al.*, 2004), [Ni(C_4_H_10_N_2_)(C_2_H_8_N_2_)_2_][B_5_O_6_(OH)_4_]_2_ (Liu *et al.*, 2006) and [Zn(dien)_2_][B_5_O_6_(OH)_4_]_2_ and [B_5_O_7_(OH)_3_Zn(tren)] (dien is diethylenetriamine and tren is tris(2-aminoethyl)amine; Wang *et al.*, 2005]. However, borates involving organic acids are scarce (Tombul *et al.*, 2007; Wu *et al.*, 2009). We describe here the synthesis and crystal structure of the title inorganic-organic hybrid borate, [Na_2_(fum)(H_3_BO_3_)(H_2_O)_4_]^.^(H_3_BO_3_) (H_2_fum is fumaric acid), (I), which represents the first one-dimensional Na^+^ coordination polymer involving both boric acid and an organic anion.

As shown in Fig. 1, the asymmetric unit of the structure of compound (I) contains two crystallographically independent Na^+^ cations (Na1 and Na2). Each Na atom is six-coordinated in the form of a distorted octahedron by two oxygen atoms (one from the carboxyl group of the fumarate (fum) anion, one from the hydroxyl group of the coordinated boric acid molecule) that occupy the axial positions, and by four water molecules in the equatorial plane. Both the fumarate anion and the coordinated boric acid act as bidentate bridging ligands to link two neighboring Na^+^ ions. The cations are again linked *via* doubly *µ*_2_-bridging water molecules [O(11) and O(12)] to generate a [Na_2_(fum)(H_3_BO_3_)(H_2_O)_4_]_n_ infinite wave-like chain running parallel to [100], with alternating Na···Na distances of 3.5942 (7) and 3.6561 (7) Å. The Na2—O4 bond length is 2.5727 (17) Å, whereas the other Na—O distances vary from 2.3606 (16) to 2.4541 (12) Å, which is in good agreement with the reported Na—O bond lengths of other Na^+^ complexes (Yi *et al.*, 2005; Huang *et al.*, 2005). The mean B—O distance of the trigonal BO_3_ groups of 1.361 (3) Å conforms with the reported B—OH distances in other boric acid adducts like [K_2_(C_4_H_2_O_4_)^.^B(OH)_3_] (1.363 (3) Å) (Tombul *et al.*, 2007), [(C_2_H_5_)_4_N^+^]_2_^.^CO_3_^2-^^.^(NH_2_)_2_CO^.^2B(OH)_3_^.^H_2_O (1.362 (2) Å), [(PPh_3_)_2_N^+^^.^Cl^-^]^.^B(OH)_3_ (1.360 (2) Å) and the 1:2 adduct of melamine with boric acid (1.362 (3) Å) (Li *et al.*, 1999; Andrews *et al.*, 1983; Roy *et al.*, 2002).

The most striking structural feature of the title compound is the oxygen-bridged one-dimensional Na infinite chain. To the best of our knowledge, no previous carboxylato-*M*BO (*M*BO is a metal borate with *M* being an alkali metal) one-dimensional coordination polymer has been reported. There is only one report about the crystal structure of a B(OH)_3_ unit bridging metal ions (Tombul *et al.*, 2007). In this example, the B(OH)_3_ molecule may be considered as coexisting with the dipotassium maleate salt. However, in structure (I), the coordinated B(OH)_3_ molecule distinctly acts as a bidentate ligand bridging two neighboring Na^+^ ions into an infinite chain, and such a coordination mode for B(OH)_3_ is unprecedented until now.

The fumarate anion, the coordinated and the free boric acid molecules are all involved in the formation of strong to medium O—H···O hydrogen bonds. From Fig. 2 it can be seen that each free B(OH)_3_ unit interacts as a donator with the fumarate ligand and the coordinated boric acid through strong O—H···O hydrogen bonding interactions [O10—H10A···O7, O8—H8A···O1, O9—H9A···O2; Table 2]. The free boric acid likewise acts as an acceptor molecule with the water and coordinated boric acid molecules as donators [O6—H6A···O8, O12—H12A···O9, O11—H11A···O10; Table 2]. Together with hydrogen bonds between the coordinated boric acid molecule and the fumarate anion [O5—H5A···O4, O7—H7···O3; Table 2] and between the water molecules and the coordinated boric acid molecule [O12—H12B···O3, O11—H11B···O2; Table 2] a three-dimensional hydrogen- bonding supported supramolecular network is generated (Fig. 3).

### Experimental

A mixture of borax (0.604 g), fumaric acid (0.234 g) and water (15 ml) was homogenized at 373 K for 1 h, producing a colourless solution. Colourless transparent block-like crystals of (I) were obtained by slow evaporation from a concentrated solution of the compound in water after standing for two weeks.

### Refinement

H atoms of the coordinated boric acid, water molecules and O8 were located in a difference Fourier map and were allowed for as riding parent atoms with *U*_iso_(H) = 1.5*U*_eq_(O) and 1.2*U*_eq_(C). Other H atoms were placed in calculated positions and were included in the refinement in the riding-model approximation, with hydroxyl O—H = 0.84 Å, methylene C—H = 0.99 Å, and with *U*_iso_(H) = 1.5*U*_eq_(O), 1.2*U*_eq_(C).

### Crystal data

**Table:**

[Na_2_(C_4_H_2_O_4_)(H_3_BO_3_)(H_2_O)_4_]·H_3_BO_3_	*F*(000) = 736
*M**_r_* = 355.77	*D*_x_ = 1.610 Mg m^−^^3^
Orthorhombic, *P**n**m**a*	Mo *K*α radiation, λ = 0.71073 Å
Hall symbol: -P 2ac 2n	Cell parameters from 10305 reflections
*a* = 14.116 (3) Å	θ = 3.1–27.4°
*b* = 6.9347 (14) Å	µ = 0.21 mm^−^^1^
*c* = 14.997 (3) Å	*T* = 295 K
*V* = 1468.1 (5) Å^3^	Block, colorless
*Z* = 4	0.39 × 0.26 × 0.25 mm

### Data collection

**Table:**

Rigaku R-AXIS RAPID diffractometer	1806 independent reflections
Radiation source: fine-focus sealed tube	1460 reflections with *I* > 2σ(*I*)
graphite	*R*_int_ = 0.023
ω scans	θ_max_ = 27.4°, θ_min_ = 3.1°
Absorption correction: multi-scan (*ABSCOR*; Higashi, 1995)	*h* = −18→18
*T*_min_ = 0.924, *T*_max_ = 0.950	*k* = −8→8
13772 measured reflections	*l* = −19→19

### Refinement

**Table:**

Refinement on *F*^2^	Primary atom site location: structure-invariant direct methods
Least-squares matrix: full	Secondary atom site location: difference Fourier map
*R*[*F*^2^ > 2σ(*F*^2^)] = 0.036	Hydrogen site location: inferred from neighbouring sites
*wR*(*F*^2^) = 0.103	H-atom parameters constrained
*S* = 1.10	*w* = 1/[σ^2^(*F*_o_^2^) + (0.0595*P*)^2^ + 0.2607*P*] where *P* = (*F*_o_^2^ + 2*F*_c_^2^)/3
1806 reflections	(Δ/σ)_max_ = 0.001
127 parameters	Δρ_max_ = 0.48 e Å^−^^3^
0 restraints	Δρ_min_ = −0.23 e Å^−^^3^

### Special details

**Table:**

Geometry. All e.s.d.'s (except the e.s.d. in the dihedral angle between two l.s. planes) are estimated using the full covariance matrix. The cell e.s.d.'s are taken into account individually in the estimation of e.s.d.'s in distances, angles and torsion angles; correlations between e.s.d.'s in cell parameters are only used when they are defined by crystal symmetry. An approximate (isotropic) treatment of cell e.s.d.'s is used for estimating e.s.d.'s involving l.s. planes.
Refinement. Refinement of *F*^2^ against ALL reflections. The weighted *R*-factor *wR* and goodness of fit *S* are based on *F*^2^, conventional *R*-factors *R* are based on *F*, with *F* set to zero for negative *F*^2^. The threshold expression of *F*^2^ > σ(*F*^2^) is used only for calculating *R*-factors(gt) *etc*. and is not relevant to the choice of reflections for refinement. *R*-factors based on *F*^2^ are statistically about twice as large as those based on *F*, and *R*- factors based on ALL data will be even larger.

### Fractional atomic coordinates and isotropic or equivalent isotropic displacement parameters (Å^2^)

**Table:**

	*x*	*y*	*z*	*U*_iso_*/*U*_eq_	
Na1	0.62506 (5)	0.2500	0.69475 (5)	0.0291 (2)	
Na2	0.37214 (5)	0.2500	0.74696 (5)	0.0329 (2)	
B1	0.54673 (15)	0.2500	0.91475 (15)	0.0306 (5)	
B2	0.34246 (15)	0.2500	1.10354 (14)	0.0272 (4)	
C1	0.58145 (12)	0.2500	0.48138 (12)	0.0239 (4)	
C2	0.48259 (12)	0.2500	0.51652 (12)	0.0285 (4)	
H2A	0.4753	0.2500	0.5795	0.034*	
C3	0.40513 (13)	0.2500	0.46889 (13)	0.0315 (4)	
H3A	0.4111	0.2500	0.4058	0.038*	
C4	0.30740 (12)	0.2500	0.50783 (13)	0.0269 (4)	
O1	0.64692 (9)	0.2500	0.53758 (9)	0.0352 (4)	
O2	0.59437 (9)	0.2500	0.39758 (8)	0.0287 (3)	
O3	0.24072 (9)	0.2500	0.45118 (9)	0.0367 (4)	
O4	0.29720 (9)	0.2500	0.59059 (9)	0.0374 (4)	
O5	0.61770 (9)	0.2500	0.85300 (10)	0.0401 (4)	
H5A	0.6759	0.2500	0.8732	0.050*	
O6	0.45594 (10)	0.2500	0.88318 (10)	0.0481 (5)	
H6A	0.4145	0.2500	0.9289	0.050*	
O7	0.56202 (8)	0.2500	1.00406 (9)	0.0402 (4)	
H7A	0.6218	0.2500	1.0174	0.050*	
O8	0.32662 (9)	0.2500	1.01491 (10)	0.0423 (4)	
H8A	0.2683	0.2500	0.9977	0.050*	
O9	0.27009 (9)	0.2500	1.16470 (9)	0.0315 (3)	
H9A	0.2178	0.2500	1.1379	0.047*	
O10	0.43302 (9)	0.2500	1.13675 (9)	0.0366 (4)	
H10A	0.4719	0.2500	1.0943	0.055*	
O11	0.49721 (7)	0.01436 (15)	0.71463 (6)	0.0354 (3)	
H11B	0.4694	−0.0581	0.6800	0.050*	
H11A	0.5038	−0.0341	0.7696	0.050*	
O12	0.75426 (7)	0.02208 (15)	0.69870 (6)	0.0358 (3)	
H12B	0.7608	−0.0362	0.6419	0.050*	
H12A	0.7415	−0.0522	0.7374	0.050*	

### Atomic displacement parameters (Å^2^)

**Table:**

	*U*^11^	*U*^22^	*U*^33^	*U*^12^	*U*^13^	*U*^23^
Na1	0.0239 (4)	0.0398 (4)	0.0238 (4)	0.000	−0.0013 (3)	0.000
Na2	0.0238 (4)	0.0429 (5)	0.0321 (5)	0.000	−0.0002 (3)	0.000
B1	0.0218 (10)	0.0481 (13)	0.0219 (10)	0.000	−0.0013 (8)	0.000
B2	0.0218 (9)	0.0366 (11)	0.0231 (10)	0.000	−0.0009 (8)	0.000
C1	0.0207 (8)	0.0298 (9)	0.0211 (8)	0.000	−0.0003 (6)	0.000
C2	0.0206 (8)	0.0431 (10)	0.0218 (9)	0.000	0.0024 (7)	0.000
C3	0.0213 (8)	0.0503 (12)	0.0230 (9)	0.000	0.0040 (7)	0.000
C4	0.0175 (8)	0.0367 (10)	0.0266 (9)	0.000	0.0022 (7)	0.000
O1	0.0185 (6)	0.0632 (10)	0.0239 (7)	0.000	−0.0020 (5)	0.000
O2	0.0210 (6)	0.0441 (8)	0.0210 (6)	0.000	−0.0005 (5)	0.000
O3	0.0186 (6)	0.0645 (10)	0.0270 (7)	0.000	0.0007 (5)	0.000
O4	0.0231 (7)	0.0661 (10)	0.0231 (7)	0.000	0.0019 (5)	0.000
O5	0.0186 (6)	0.0795 (11)	0.0223 (7)	0.000	−0.0008 (5)	0.000
O6	0.0194 (7)	0.1019 (14)	0.0229 (7)	0.000	−0.0010 (5)	0.000
O7	0.0175 (6)	0.0822 (12)	0.0208 (7)	0.000	−0.0013 (5)	0.000
O8	0.0172 (6)	0.0852 (11)	0.0243 (7)	0.000	−0.0002 (5)	0.000
O9	0.0206 (6)	0.0504 (8)	0.0235 (7)	0.000	−0.0002 (5)	0.000
O10	0.0206 (6)	0.0642 (10)	0.0248 (7)	0.000	−0.0012 (5)	0.000
O11	0.0378 (5)	0.0379 (5)	0.0303 (5)	−0.0042 (4)	−0.0043 (4)	−0.0039 (4)
O12	0.0384 (5)	0.0371 (5)	0.0318 (5)	−0.0006 (4)	0.0005 (4)	0.0004 (4)

### Geometric parameters (Å, °)

**Table:**

Na1—O5	2.3756 (17)	C1—O1	1.251 (2)
Na1—O1	2.3771 (17)	C1—O2	1.270 (2)
Na1—O12^i^	2.4140 (12)	C1—C2	1.492 (2)
Na1—O12	2.4140 (12)	C2—C3	1.306 (3)
Na1—O11	2.4529 (12)	C2—H2A	0.9500
Na1—O11^i^	2.4529 (12)	C3—C4	1.498 (2)
Na1—Na2^ii^	3.5955 (12)	C3—H3A	0.9500
Na1—Na2	3.6552 (12)	C4—O4	1.250 (2)
Na2—O6	2.3606 (16)	C4—O3	1.268 (2)
Na2—O12^iii^	2.4353 (12)	O5—H5A	0.8756
Na2—O12^iv^	2.4353 (12)	O6—H6A	0.9013
Na2—O11^i^	2.4541 (12)	O7—H7A	0.8673
Na2—O11	2.4541 (12)	O8—H8A	0.8627
Na2—O4	2.5727 (17)	O9—H9A	0.8400
Na2—Na1^iii^	3.5955 (12)	O10—H10A	0.8400
B1—O7	1.357 (3)	O11—H11B	0.8224
B1—O5	1.364 (3)	O11—H11A	0.8951
B1—O6	1.366 (3)	O12—Na2^ii^	2.4353 (12)
B2—O8	1.348 (3)	O12—H12B	0.9473
B2—O10	1.372 (2)	O12—H12A	0.7967
B2—O9	1.373 (2)		
			
O5—Na1—O1	175.05 (6)	O1—C1—O2	124.12 (17)
O5—Na1—O12^i^	90.49 (4)	O1—C1—C2	116.94 (17)
O1—Na1—O12^i^	85.77 (4)	O2—C1—C2	118.94 (16)
O5—Na1—O12	90.49 (4)	C3—C2—C1	126.15 (18)
O1—Na1—O12	85.77 (4)	C3—C2—H2A	116.9
O12^i^—Na1—O12	81.80 (5)	C1—C2—H2A	116.9
O5—Na1—O11	81.16 (4)	C2—C3—C4	123.90 (18)
O1—Na1—O11	102.48 (4)	C2—C3—H3A	118.1
O12^i^—Na1—O11	171.52 (5)	C4—C3—H3A	118.1
O12—Na1—O11	96.70 (4)	O4—C4—O3	125.46 (16)
O5—Na1—O11^i^	81.16 (4)	O4—C4—C3	119.56 (16)
O1—Na1—O11^i^	102.48 (4)	O3—C4—C3	114.98 (16)
O12^i^—Na1—O11^i^	96.70 (4)	C1—O1—Na1	124.91 (12)
O12—Na1—O11^i^	171.52 (5)	C4—O4—Na2	149.11 (12)
O11—Na1—O11^i^	83.55 (5)	B1—O5—Na1	135.26 (12)
O6—Na2—O12^iii^	93.03 (4)	B1—O5—H5A	117.0
O6—Na2—O12^iv^	93.03 (4)	Na1—O5—H5A	107.7
O12^iii^—Na2—O12^iv^	80.93 (5)	B1—O6—Na2	140.35 (13)
O6—Na2—O11^i^	79.08 (4)	B1—O6—H6A	110.2
O12^iii^—Na2—O11^i^	171.82 (5)	Na2—O6—H6A	109.5
O12^iv^—Na2—O11^i^	97.21 (4)	B1—O7—H7A	112.5
O6—Na2—O11	79.08 (4)	B2—O8—H8A	117.0
O12^iii^—Na2—O11	97.21 (4)	B2—O9—H9A	109.5
O12^iv^—Na2—O11	171.82 (5)	B2—O10—H10A	109.5
O11^i^—Na2—O11	83.50 (5)	Na1—O11—Na2	96.30 (4)
O6—Na2—O4	174.20 (6)	Na1—O11—H11B	133.0
O12^iii^—Na2—O4	91.38 (4)	Na2—O11—H11B	100.9
O12^iv^—Na2—O4	91.38 (4)	Na1—O11—H11A	106.6
O11^i^—Na2—O4	96.64 (4)	Na2—O11—H11A	98.2
O11—Na2—O4	96.64 (4)	H11B—O11—H11A	113.7
O7—B1—O5	123.60 (18)	Na1—O12—Na2^ii^	95.71 (4)
O7—B1—O6	119.43 (18)	Na1—O12—H12B	109.3
O5—B1—O6	116.97 (18)	Na2^ii^—O12—H12B	120.7
O8—B2—O10	120.84 (18)	Na1—O12—H12A	105.7
O8—B2—O9	122.36 (17)	Na2^ii^—O12—H12A	109.3
O10—B2—O9	116.80 (17)	H12B—O12—H12A	113.7

Symmetry codes: (i) *x*, −*y*+1/2, *z*; (ii) *x*+1/2, *y*, −*z*+3/2; (iii) *x*−1/2, *y*, −*z*+3/2; (iv) *x*−1/2, −*y*+1/2, −*z*+3/2.

### Hydrogen-bond geometry (Å, °)

**Table:**

*D*—H···*A*	*D*—H	H···*A*	*D*···*A*	*D*—H···*A*
O6—H6A···O8	0.90	1.79	2.690 (2)	177
O10—H10A···O7	0.84	1.86	2.6973 (19)	178
O5—H5A···O4^ii^	0.88	1.80	2.6713 (19)	177
O7—H7A···O3^ii^	0.87	1.74	2.6104 (18)	178
O12—H12B···O3^v^	0.95	2.04	2.9355 (15)	157.8
O12—H12A···O9^vi^	0.80	2.02	2.8063 (14)	171.5
O11—H11B···O2^v^	0.82	1.98	2.8042 (14)	175.5
O11—H11A···O10^vi^	0.90	2.24	3.0494 (15)	150.5
O8—H8A···O1^iii^	0.86	1.79	2.6559 (19)	180
O9—H9A···O2^iii^	0.84	1.82	2.6504 (19)	168

Symmetry codes: (ii) *x*+1/2, *y*, −*z*+3/2; (v) −*x*+1, −*y*, −*z*+1; (vi) −*x*+1, −*y*, −*z*+2; (iii) *x*−1/2, *y*, −*z*+3/2.
